# Effectiveness of Percutaneous Coronary Intervention within 12 Hours to 28 Days of ST-Elevation Myocardial Infarction in a Real-World Chinese Population

**DOI:** 10.1371/journal.pone.0058382

**Published:** 2013-03-12

**Authors:** Xingli Wu, Dingyou Yang, Yusheng Zhao, Caiyi Lu, Yu Wang

**Affiliations:** 1 Institute of Geriatric Cardiology, China PLA General Hospital, Beijing, China; 2 Department of Traditional Chinese Medicine, the First Affiliate Hospital of China PLA General Hospital, Beijing, China; Universityhospital Düsseldorf, Germany

## Abstract

**Objectives:**

Percutaneous coronary intervention( PCI) for ST-elevation myocardial infarction (STEMI) has been widely accepted for patient who come within 12 hours, but for those who come to the hospital late (12 hours to 28 days) the long-term data and possible predictors are limited regarding ‘hard’ endpoints in ‘real world’.

**Methods:**

The registry data of all 5523 consecutive patients admitted due to an incident STEMI (12 hours to 28 days) in our center were analyzed. Patients were divided into 3 age groups (age<65; age = 65–74; age ≥75) and two therapeutic groups including conservative and PCI group. The primary endpoints included 30-day mortality and 1-year mortality.

**Results:**

The clinical characteristics include female gender; history of diabetes mellitus, previous myocardial infarction, cerebral vascular disease, chronic renal failure, atrial fibrillation, hypertension, anemia, gastric bleeding; presentation of ventricular tachycardia/ventricular fibrillation, pneumonia, heart failure, multiple organ failure and cardiogenic shock. The ratio of all the above factors increased with the age getting older (all p<0.05), while that of the PCI decreased significantly with ageing (53.9%, 36.3% and 21.7%). Except hypertension, all the other factors were less seen in the PCI group than in the conservative group (p<0.01). Pooled estimates, based on type of therapy and age groups, PCI resulted in significantly lower 30-day and 1-year mortality. Cox analysis showed the positive predictors for 30 days and 1 year mortality were heart failure, cerebral vascular disease, chronic renal failure, ventricular tachycardia/ventricular fibrillation, age, female, gastric intestinal bleeding, cardiogenic shock, multiple organ failure, while PCI was a negative predictor. ROCs analysis showed AUCs were always higher for PCI group.

**Conclusions:**

The elderly have more comorbidities and higher rates of mortality, mandating thorough evaluation before acceptance for PCI. PCI between 12 hours to 28 days in all ages of patients including the elderly with STEMI is significantly more effective than conservative therapy.

## Introduction

With the rapidly increasing proportion of the elderly worldwide, more people are living with ST-elevation myocardial infarction (STEMI), and percutaneous coronary intervention (PCI) for coronary revascularization is becoming more common [1]–[3]. Because clinical trials frequently exclude elderly patients, the management strategy for this age group remains a major therapeutic challenge. It appears that elderly patients are treated more conservatively than younger ones due to the higher incidence of contraindications and delayed time windows for PCI [4]. Some evidence-based data shows that the elderly have the potential to gain the most clinical benefit from an early invasive approach because of their higher baseline risk [5], while other studies have showed reduced success and increased or unchanged adverse outcomes after PCI in the elderly, even in the stenting eras [6], [7].

Based on several large clinical trials performed in recent years, the 2010 ESC/EACTS and 2011 ACCF/AHA/SCAI guidelines on STEMI revascularization have recommended both primary PCI within 12 h and PCI 3–24 h after fibrinolysis, regardless of whether the culprit vessel is opened or not [8]–[11]. These guidelines also state that patients presenting between 3 and 28 days with persistent coronary artery occlusion without ongoing chest pain or inducible ischemia do not benefit from PCI, while limited data indicates controversially that in strictly selected patient subgroups, PCI after more than 24 hours or 3 days of STEMI holds an equal, less or greater risk than just medical therapy alone [11]–[14].

A wealth of data generally supports an early invasive strategy over a conservative strategy in STEMI, however, there is currently a paucity of data pertaining to the safety and efficacy of delayed or selected PCI between 12 hours and 28 days after onset of STEMI in unselected populations [15]–[16]. Thus, there may be great practical value in investigating the risk/benefits ratio of delayed PCI in STEMI, especially among the elderly, since this subgroup has not been well-represented in these trials. Our cohort analysis aimed to investigate clinical characteristics and outcomes in patients receiving PCI 12 hours to 28 days from a large consecutive sample with STEMI as compared with those receiving conservative therapy and to determine predictors for short term and long term mortalities.

## Methods

### Study Population

A total of 5523 consecutive patients admitted due to an incident STEMI to the Chinese PLA General Hospital between 1996 and 2010 were registered and analyzed. Patients were divided into three age groups (age <65; age 65–74; age ≥75) and two therapeutic groups (conservative and PCI). Sixteen basic clinical variables were recorded: age; female gender; history of diabetes mellitus (DM), previous myocardial infarction (OMI), cerebral vascular disease (CVD), chronic renal failure (CRF), atrial fibrillation (AF), hypertension, anemia, and gastric bleeding (GIB); presentation of ventricular tachycardia/ ventricular fibrillation (VT/VF), heart failure (HF), pneumonia, cardiogenic shock, and multiple organ failure (MOF); and PCI. Written informed consent was obtained from all patients and the protocol was reviewed and approved by the Chinese PLA General Hospital Medical Ethics Committee.

### Therapeutic Protocol

Patients in the conservative group were treated by thrombolysis (2%), antiplatelet agents, angiotensin-converting enzyme inhibitor, β-blocker, nitrates, statins, unfractionated heparin or low-molecular-weight heparin, unless contraindicated.

The PCI group consisted of all patients on whom the intervention procedure was conducted 12 hours to 28 days after the onset of chest pain . Before Dec. 2000, all patients were treated with aspirin 300 mg orally as the loading dosage and then 100 mg daily. Since then, additional clopidogrel 300 mg loading dose, followed by 75 mg per day was administered for at least 12 month. In the catherization laboratory, all patients were given unfractionated heparin 100 U/kg body weight at the beginning of the procedure and the anticoagulation level was adjusted to maintain activated partial thromboplastin time (APTT) 2 to 3 times of the normal value during the procedure. After PCI, low weight heparin was used for 3 to 5 days [17]. PCI, including balloon angioplasty and/or stent implantation, was performed for the culprit artery with a Thrombolysis In Myocardial Infarction (TIMI) flow grade ≤ 2, and a successful procedure was defined as infarcted artery stenosis < 30% associated with a TIMI flow grade of 2 or 3.

### Definitions

STEMI was defined as ischemic chest discomfort lasting at least 30 minutes and associated with ≥1 mm ST segment elevation in ≥2 contiguous leads or a presumed new-onset left bundle branch block, with either a rise in troponin or creatine kinase MB of more than three times the upper limit of normal.

Heart failure was defined by Killips class ≥II. Gastric bleeding was defined by hematemesis or hemafecia with a drop in hemoglobin of >30 g/L and/or requiring transfusion. Chronic renal failure was defined by an increase of serum creatinine to >200 mmol/L (2.27 g/L). Stroke was defined by the onset of persistent loss of neurological function caused by an ischemic or hemorrhagic event. Cardiogenic shock was defined by hypotension (systolic BP <90 mmHg for at least 30 min or needing supportive measures), evidence of end-organ hypoperfusion, or a cardiac index of < 2.2 L/(min.m^2^) and pulmonary capillary wedge pressure of ≥18 mmHg. Anemia was defined by a lower hemoglobin level of <90 g/L. Pneumonia was diagnosed according to the recommendations of the CDC [18]. Multiple organ failure (MOF) was defined as failure of two or more organs using the modified Goris’s MOF scoring system [19].

### Study End-points and Follow-up

Clinical follow-up was completed through telephone conversation or outpatient clinical interview at 30 days and one year after discharge. All the patients completed the follow-up. The primary end-point was all-cause death.

### Statistical Analysis

Continuous variables are presented as mean ± SD and categorical variables as a percentage. Categorical variables were compared using an X^2^ test and continuous variables using the Student T test. Univariate and multivariate Cox regression analyses were used to identify independent predictors of 30-day and one-year mortality. The final results are presented as hazard ratios (HRs) with associated 95% confidence intervals (CIs). In the Cox regression analyses, the 16 variables listed were entered into the model (age, female gender, hypertension, DM, OMI, HF, CVD, pneumonia, CRF, anemia, GIB, AF, VT/VF, cardiogenic shock, MOF, and PCI). Cumulative hazard curves were constructed according to age group and therapy. The predictive value of PCI for mortality was detected by plotting receiver-operating-characteristic (ROC) curves for the 16 baseline covariates with and without PCI. Analysis was performed using SPSS (SPSS Inc, Chicago, IL) for Windows, version 19.0. A *P*-value <.05 was regarded as significant.

## Results

### Baseline Clinical Characteristics

The ratio of all the (non-PCI) clinical characteristics factors (female, DM, CVD, CRF, AF, VT/VF, hypertension, pneumonia, anemia, GIB, cardiogenic shock, and MOF) increased (all *P*<.001) with age as specified (age <65; age = 65–74; age ≥75); while that of the PCI decreased significantly (*P*<.001) (**Table 1**).

**Table 1:** Baseline Characteristic of Different Age Groups (n = 5523).

Variable	Age <65	Age = 65–74	Age ≥75	?^2^	*P* value
Patients, n (%)	2720 (49.3)	1538 (27.9)	1265 (22.9)		–
Age, mean (SD), y	51.9±8.7	69.5±2.9	80.1±4.4	8791.02#	.000
Female (%)	15.7	25.6	24.3	73.966	.000
DM (%)	19.0	26.3	29.7	63.816	.000
Hypertension (%)	39.8	57.0	69.5	197.2	.000
OMI (%)	9.3	18.1	28.7	244.6	.000
HF (%)	19.3	23.1	30.4	60.267	.000
CVD (%)	1.9	4.2	7.8	79.621	.000
Pneumonia (%)	3.1	8.6	20.5	334.2	.000
CRF (%)	4.8	12.4	22.3	277.9	.000
Anemia (%)	8.9	24.6	38.9	506.8	.000
GIB (%)	0.3	1.2	2.7	43.547	.000
AF (%)	6.6	6.2	8.4	6.076	.048
VT/VF (%)	0.8	1.6	2.4	16.216	.000
Cardiogenic shock (%)	2.2	3.4	6.7	52.577	.000
PCI (%)	53.9	36.3	21.7	394.1	.000
MOF (%)	0.2	1.1	3.9	84.21	.000

DM, diabetes mellitus; OMI, previous myocardial infarction; HF, heart failure (Killip class ≥II) ; CVD, cereberal vascular disease; CRF, chronic renal failure; GIB, Gastric bleeding; AF, atrial fibrillation; VT/VF, ventricular tachycardia / ventricular fibrillation; MOF, multiple organ failure; PCI, percutaneous coronary intervention.

# F value of levene test.

Based on therapeutic strategy, all the above clinical characteristics (except hypertension) were observed less in the PCI group than the conservative group (all *P*<.001) (**Table 2**).

**Table 2:** Baseline Characteristic of PCI and Conservative Therapy (n = 5523).

Variable	PCI	conservative	?^2^	*P*
Patients, n (%)	2299 (41.6)	3224 (58.4)	–	–
Age, mean (SD), y	59.1±12.2	66.1±13.2	17.693#	0.000
Female (%)	15.7	23.8	53.399	0.000
Diabetes mellitus (%)	21.8	24.7	6.403	0.011
Hypertension (%)	49.8	48	0.305	0.581
OMI (%)	9.4	21.1	135.1	0.000
HF (%)	16	27.9	107.4	0.000
CVD (%)	1.5	5.6	58.51	0.000
Pneumonia (%)	3.1	12.5	150.5	0.000
CRF (%)	6.3	14.2	86.642	0.000
Anemia (%)	15.3	23.6	58.004	0.000
GIB (%)	0.4	1.7	19.604	0.000
AF (%)	2.3	10.2	131.1	0.000
VT/VF (%)	0.5	2	21.794	0.000
Cardiogenic shock (%)	1.6	5	45.241	0.000
MOF (%)	0.3	2	35.87	0.000

# F value of levene test; Abbreviations are the same as in table 1.

### Short-term and Long-term Outcomes

PCI was performed on 41.6% of patients and conservative therapy used for the remaining 58.4%.Angiographic success among the three age groups (age <65; age = 65–74; age ≥75) was obtained at 99.8%, 99.6% and 99.4%, respectively; there was a generalized incremental trend in 30-day mortality and 1-year mortality (all P <.001) (**Table 3**). Pooled estimates of overall 30-day and one-year mortality were 2.2% for PCI vs 14.2% for conservative and 2.7% for PCI vs 17.6% for conservative, respectively (**Table 4**). For all age groups and both mortality measures, the PCI results were significantly better than the conservative (all *P*< .001) (**Table 5**).

**Table 3:** Mortality at 30 Days and 1 Year of Different Therapy in 3 Age Groups.

	Age <65				Age 65–74				Age ≥75			
	PCI	non–PCI	?^2^	*P*	PCI	non-PCI	?^2^	*P*	PCI	non-PCI	?^2^	*P*
	n = 1467	n = 1253			n = 559	n = 979			n = 274	n = 991		
30-d, n (%)	13 (0.9)	89 (7.1)	72.36	0.000	18 (3.2)	137 (14.0)	45.37	0.000	19 (6.9)	230 (23.3)	36.11	0.000
1-y, n (%)	20 (1.4)	108 (8.6)	79.34	0.000	23 (4.1)	158 (16.1)	49.31	0.000	20 (7.3)	301 (30.4)	60.58	0.000

**Table 4:** Mortality at 30 Days and 1 Year of Different Age Groups.

	Age <65	Age 65–74	Age ≥75		
	n = 2720	n = 1538	n = 1265	?^2^	*P*
30-d, n (%)	102 (3.8)	155 (10.1)	249 (19.7)	266.2	0.000
1-y, n (%)	128 (4.7)	181 (11.8)	321 (25.4)	366.2	0.000

**Table 5:** Mortality at 30 Days and 1 Year of Different Therapy.

	PCI	Conservative		
	n = 2299	n = 3224	?^2^	*P*
30-d, n (%)	50 (2.2)	457 (14.2)	231.8	0.000
1-y, n (%)	63 (2.7)	568 (17.6)	293.5	0.000

The ratio of cases underwent PCI increased with time, which were 10.4%,35.4% and 57.5% separately in the year 1996 to 2000, 2001 to 2005 and 2006 to 2010. No death occurred in all the 104 patients who underwent PCI in 1996–2000, and there was no significant change in the rate of mortality, stroke and GIB among different times (**Table 6**).

**Table 6:** The ratio of mortality, GIB, stroke and PCI in different time periods.

	1996–2000	2001–2005	2006–2010	?^2^	*P*
**30 days mortality**					
Non-PCI	123 (13.7%)	158 (13.2%)	177 (15.5%)	2.728	0.256
PCI	0 (0%)	16 (2.5%)	34 (2.25%)	2.55	0.279
**1 year mortality**					
Non-PCI	156 (17.4%)	196 (16.4%)	297 (19.1%)	2.794	0.247
PCI	158 (15.8%)	214 (11.6%)	260 (9.7%)	0.278	0.870
**GIB**					
Non-PCI	9 (1%)	18 (1.5%)	28 (2.5%)	6.745	0.034
PCI	0 (0%)	3 (0.5%)	8 (0.5%)	0.56	0.756
**Stroke**					
Non-PCI	33 (3.7%)	51 (4.3%)	97 (8.5%)	28.438	0.000
PCI	1 (1%)	6 (0.9%)	32 (2.1%)	4.059	0.131
**Rate of PCI**					
Non-PCI	895 (89.6%)	1193 (64.6%)	1139 (42.5%)	706.566	0.000
PCI	104 (10.4%)	653 (35.4%)	1538 (57.5%)		

The differences among time periods were analyzed by using pearson **χ** ^2^.

### Predictors of Mortality at 30 Days and 1 Year

Univariable Cox regression analysis showed that PCI was a negative predictor for 30-day and one-year mortality, hypertension was a positive predictor only for one-year mortality, and all other 14 variables were significantly positive predictors for both 30-day and one-year mortality (**Table 7**).

**Table 7:** Univariate Predictors of 30 Days and 1 Year Mortality by Cox Regression Analysis.

	30-d mortality		1-y mortality	
Predictor	HR (95% CI)	*P*	HR (95% CI)	*P*
Age1	2.897 (2.239–3.750)	0.000	2.701 (2.134–3.419)	0.000
Age2	6.322 (4.966–8.048)	0.000	6.915 (5.561–8.598)	0.000
Female	1.672 (1.364–2.051)	0.000	1.470 (1.215–10779)	0.000
OMI	2.119 (1.718–2.613)	0.000	1.051 (1.231–1.853)	0.000
Hypertension	1.153 (0.961–1.385)	0.126	1.186 (1.004–1.400)	0.044
DM	1.503 (1.231–1.835)	0.000	1.459 (1.215–1.752)	0.000
AF	1.670 (1.230–2.267)	0.000	1.808 (1.373–2.381)	0.000
CVD	3.512 (2.538–4.860)	0.000	3.835 (2.836–5.185)	0.000
CRF	4.209 (3.400–5.212)	0.000	3.869 (3.164–4.731)	0.000
HF	2.341 (1.934–2.833)	0.000	2.155 (1.807–2.569)	0.000
Pneumonia	3.991 (3.166–5.033)	0.000	4.977 (4.025–6.155)	0.000
GIB	5.898 (3.502–9.934)	0.000	6.444 (3.892–10.668)	0.000
Anemia	2.194 (1.801–2.672)	0.000	9.895 (6.268–15.623)	0.000
Cardiogenic shock	15.295 (11.344–20.622)	0.000	14.881 (11.008–20.116)	0.000
PCI	0.137 (0.102–0.184)	0.000	0.132 (0.101–0.172)	0.000
VT/VF	9.770 (6.187–15.430)	0.000	9.895 (6.268–15.623	0.000
MOF	10.234 (6.366–16.452)	0.000	21.42 (12.68–36.18)	0.000

Age1, cohort age< 65 and age 65–74; Age2, cohort age < 65 and age > = 75; HR, Hazard ratio; CI, confidence interval; other abbreviations seen in table 1.

Multivariate Cox regression analysis demonstrated that the significantly positive predictors (*P*<.05) for 30-day and one-year mortality were HF, CVD, CRF, VT/VF, age, female, GIB, cardiogenic shock, and MOF, while pneumonia was a positive predictor only for one-year mortality. After adjusting for age and all other factors listed (above), PCI was found to be a negative predictor for both 30-day mortality (HR, 0.282; 95% confidence interval, 0.209–0.379; *P*<.001) and one-year mortality (HR, 0.311; 95% confidence interval, 0.238–0.407; *P*<.001). OMI was associated only with a (remarkably) reduced one-year mortality *(P*<.001) (**Table 8**).

**Table 8:** Multivariate Predictors of 30 Days and 1 Year Mortality by Cox Analysis.

	30-d mortality		1-y mortality	
Predictor	HR (95% CI)	*P*	HR (95% CI)	*P*
Age	1.445 (1.287–1.623)	0.000	1.401 (1.260–1.559)	0.000
Female	1.515 (1.246–1.843)	0.000	1.448 (1.207–1.737)	0.000
OMI	0.616 (0.504–0.754)	0.000		
HF	1.314 (1.093–1.580)	0.004	1.271 (1.0731.507)	0.006
CVD	1.436 (1.074–1.919)	0.014	1.446 (1.115–1.875)	0.005
Pneumonia	1.400 (1.155–1.697)	0.001		
CRF	1.936 (1.472–2.153)	0.000	1.780 (1.472–2.153)	0.000
GIB	1.815 (1.178–2.797)	0.007	1.915 (1.292–2.837)	0.001
VT/VF	2.674 (1.893–3.778)	0.000	3.021 (2.187–4.173)	0.001
Cardiogenic shock	5.734 (4.599–7.150)	0.000	5.760 (4.675–7.097)	0.000
PCI	0.282 (.209–.379)	0.000	0.311 (.238–.407)	0.000
MOF	1.938 (1.356–2.769)	0.000	2.065 (1.526–2.794)	0.000

Variables entered the Cox analyses are Age; Gender; Hypertension; DM, OMI, HF, CVD, Pneumonia; CRF, Anemia; GIB; AF, VT/VF, Cardiogenic shock; MOF; PCI. Variable removed from the analyses of 30 days mortality are AF, anemia, DM, hypertension, pneumonia and OMI. Variable removed from the analyses of 1 year mortality are AF, anemia, DM, hypertension.

**Figure 1:**
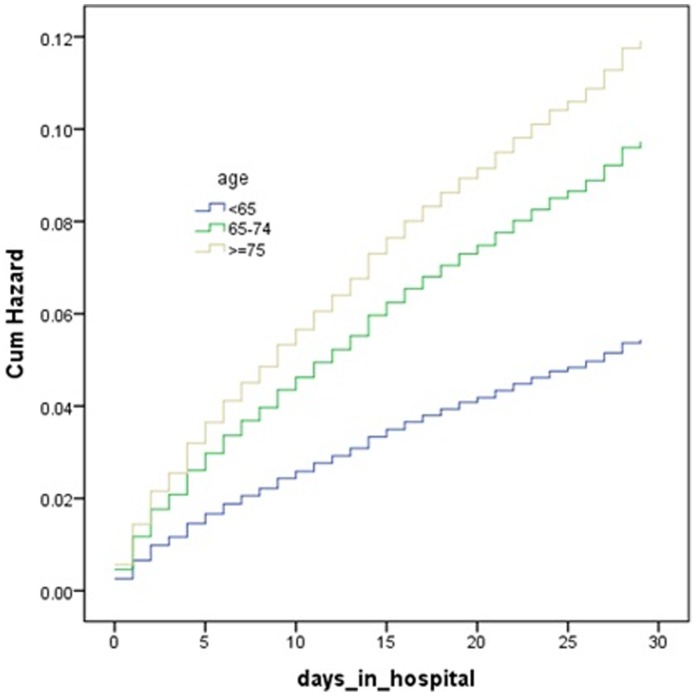
Predicted cumulative hazard of 30 days mortality in different age groups of patients. The curves were constructed according to the age groups with the use of Cox regression analysis.

**Figure 2:**
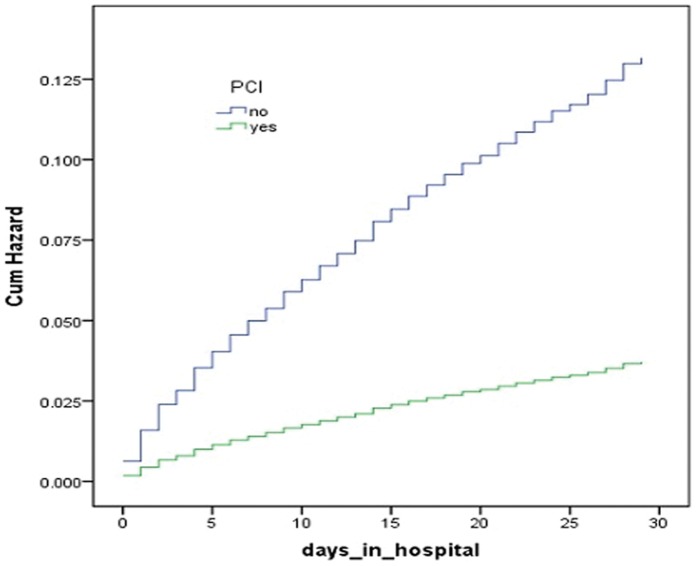
Predicted cumulative hazard function of 30 days mortality for different therapies. The curves were constructed according to the PCI or conservative therapie with the use of Cox regression analysis.

**Figure 3:**
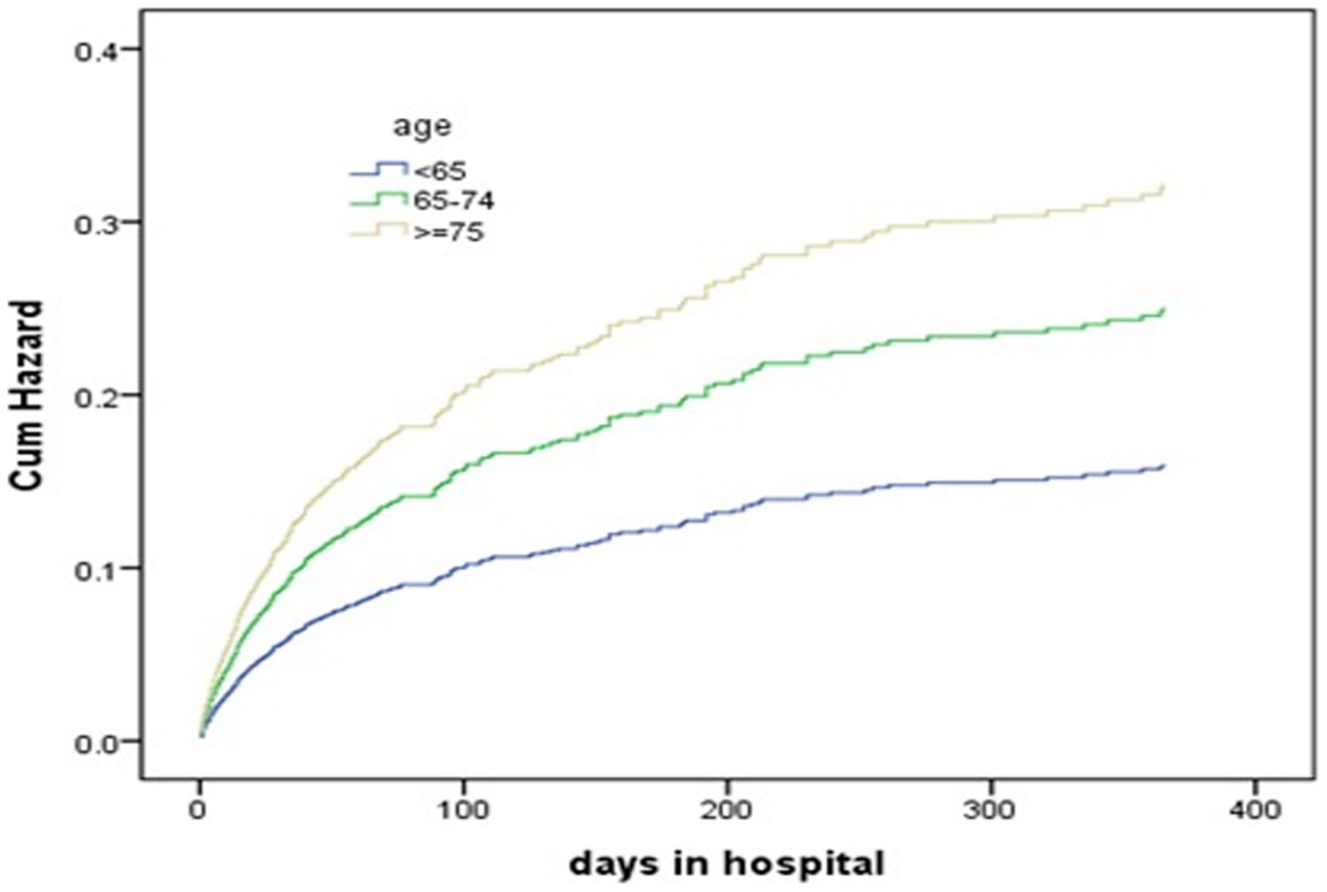
Predicted cumulative hazard function of 1 year mortality for different age groups of patients. The curves were constructed according to the age groups with the use of Cox regression analysis.

**Figure 4:**
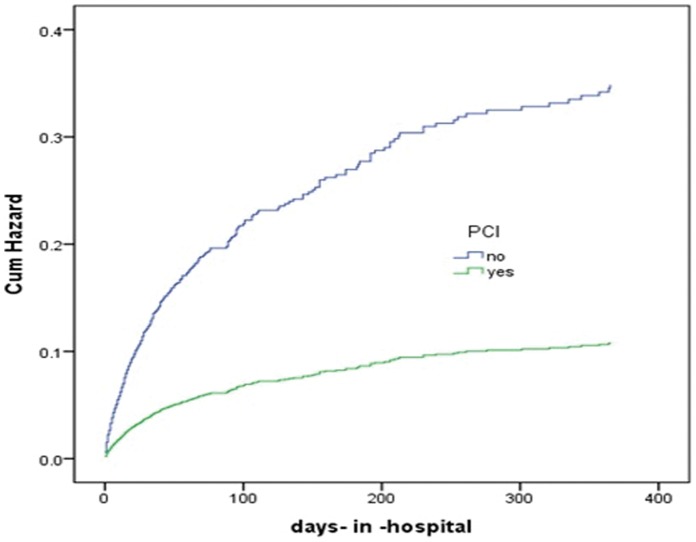
Predicted cumulative hazard function of 1 year mortality for different therapies. The curves were constructed according to the PCI or conservative therapy with the use of Cox regression analysis.

Cumulative hazard curves were constructed according to PCI and age groups with the use of Cox regression analysis, and showed these were (all) strong predictors for both 30-day and 1-year mortality (**Fig. 1** **,** **Fig. 2** **,** **Fig. 3** **,** **Fig. 4**).

**Figure 5:**
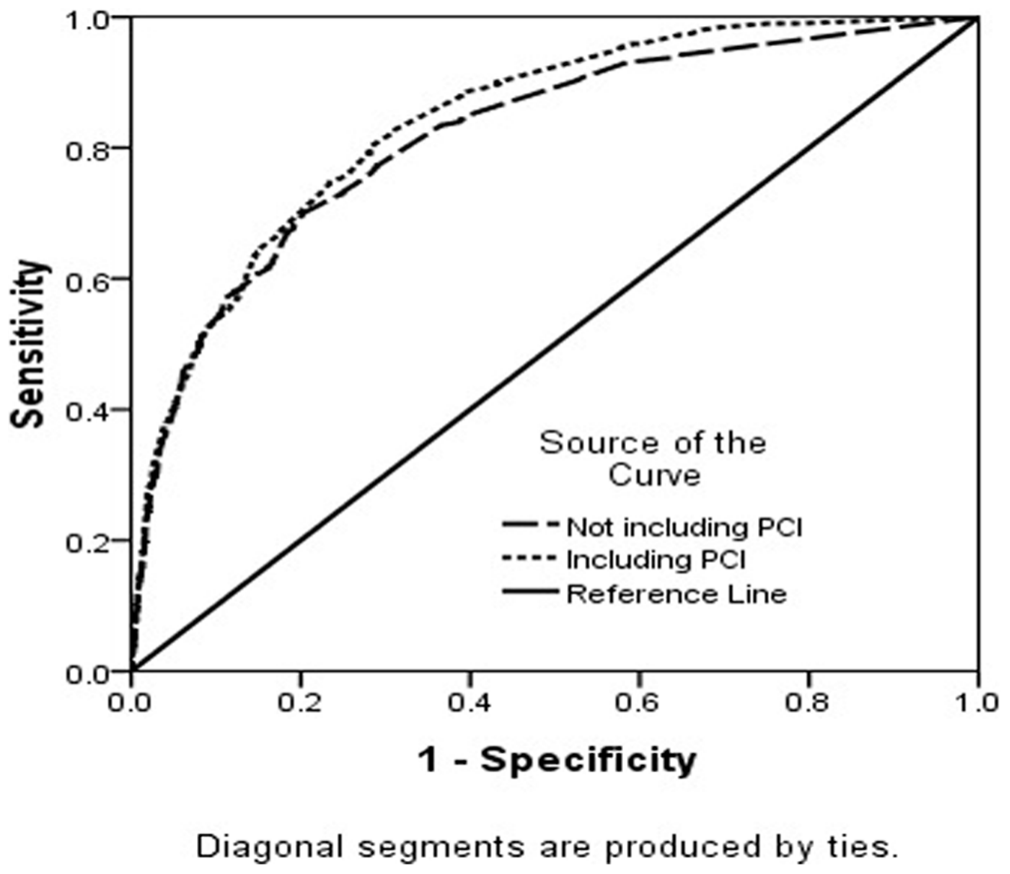
Receiver-operator characteristic curves (ROCs) of the predicting performance of PCI on 30 days mortality. The curves are based on risk-prediction models incorporating 16 clinical covariates that either include the PCI or did not include PCI. The area under the ROC curve (AUC) for mortalities was 0.841(0.824–0.858) and 0.819(0.799–0.839) for the risk model with and without the PCI separately (both *P*<0.001).

**Figure 6:**
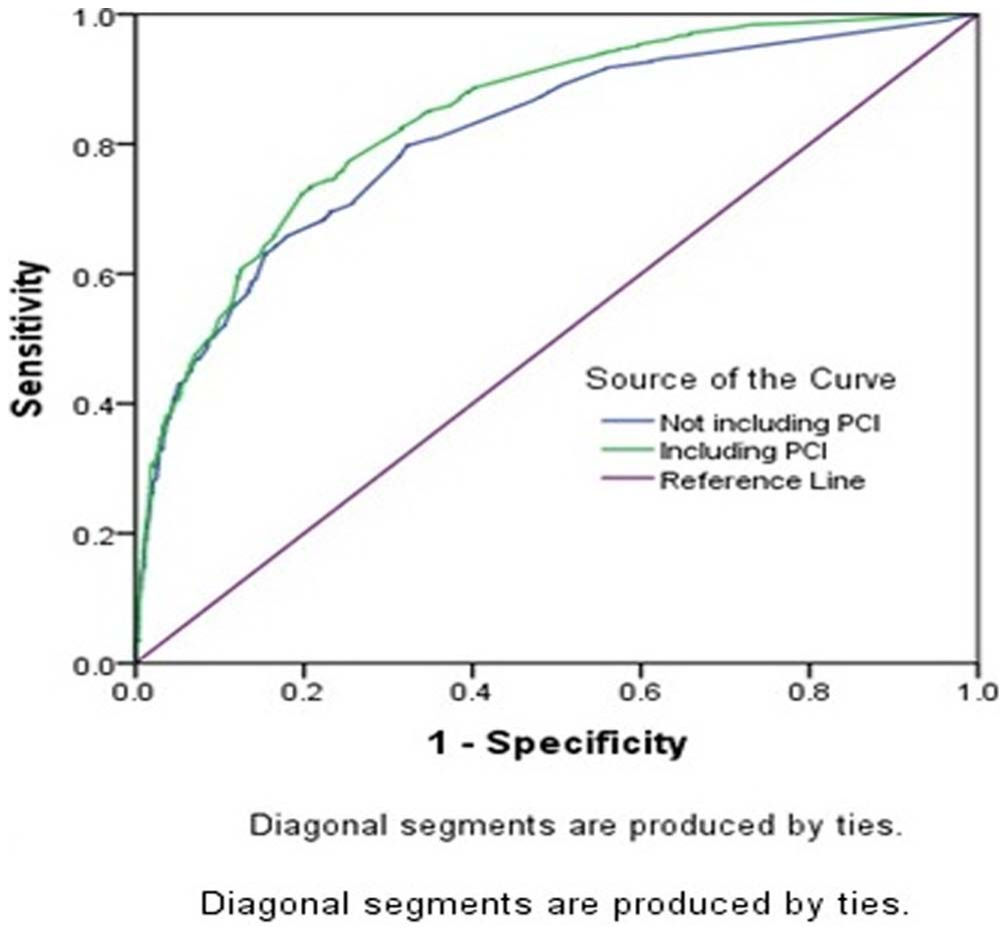
Receiver-operator characteristic curves (ROCs) of the predicting performance of PCI on 1 year mortality. The curves are constructed as in Fig. 1. The AUC for mortalities was 0.841(0.825–0.857) and 0.811(0.792–.0830) for the risk model with and without the PCI separately (both *P*<0.001).

We constructed ROC curves for the model incorporating the 16 risk factors with and without inclusion of PCI, using mortality at 30 days and one year as the outcome (**Fig. 5**, **Fig**. **6**). The area under the ROC curve (AUC) for mortality at 30 days and one year was 0.841 (0.824–0.858) and 0.819 (0.799–0.839) for the risk model with and without the PCI (both *P*<.001), respectively, while the AUC for mortality at one year was 0.841(0.825–0.857) and 0.811(0.792–0.830) for the risk model with and without the PCI, respectively (both *P*<.001).

## Discussion

It has been accepted that in the general population, primary PCI in STEMI patients within 6–12 h and possibly up to 60 h from symptom onset has shown improved clinical outcome [8], [20], [21]. The data pertaining to the safety and efficacy of PCI between 12 h to 28 days after onset of STEMI are limited, however, and results remain controversial, especially for the elderly [13]–[15], [22]–[26]. This study has provided evidence within these parameters.

### Baseline Clinical Characteristics

In this study, PCI was performed in 41.6% of patients; the subjects enrolled in the conservative group were proportionately more female, much older, and had a far higher incidence of comorbidity, which is partly consistent with those in the Italian Registry and Melbourne octogenarian studies [25]–[27].

### 30-Day and One-Year Outcomes

Our major findings were that both 30-day mortality and one-year mortality rates increased with age and that PCI improved the outcome significantly in all specified age groups when carried out within 12 hours to 28 days of STEMI onset.

In this study, the technical and angiographic success rate of PCI was more than 99.4% even for patients over 75 years old. The total average 30-day mortality was 2.2% for PCI and 14.2% for conservative therapy groups, while one- year mortality was 2.7% vs 17.6%, which was consistent with mortality rates of 4% to 8% as reported in other studies [7], [28]. The beneficial outcome of PCI as compared with conservative therapy was also demonstrated in different age groups.

The all-cause mortality rate in primary PCI for octogenarians was lower than that reported elsewhere. Here, the 12-month mortality rates for age <80 years vs. octogenarians were 29% vs 41% and the 30-day 17% vs 26%, respectively [22]. However, the 30-day mortality rate was higher than the six PCI registries, in which in-hospital mortality rates ranged from 1.5% to 5.2% across databases, with an average of 3.0% for patients aged 75 years [29]. Also, the one-year mortality rate in the age ≥75 group was markedly lower in the PCI group than that of 51.4% as reported by de Labriolle, but higher than the 19.3% reported by Zeyme–in the case of the latter, this may be due to the shorter follow-up duration [30], [31].

Regarding the delayed time window of PCI for STEMI, these results support PCI. In a randomized study involving 2166 stable patients who had total occlusion of the infarct-related artery three to 28 days after myocardial infarction and who met a high-risk criterion (an ejection fraction of <50% or proximal occlusion), PCI did not reduce the occurrence of death during four years of follow-up [14]. However, Yazici found no significant differences here between the two PCI groups early (≤3 days) and late (>3 days) [15], while Yip reported that the performance of PCI on >4 days was safe and had a relative low mortality rate during the long-term follow-up [16]. Our findings coincide with the latter two studies [15], [16].

Although the ratio of cases underwent PCI in this series increased with time (1996 to 2000, 2001 to 2005 and 2006 to 2010), there were no significant difference in the 30 day and 1 year mortality among the three year periods for both PCI and conservative groups. This may reflected more carefully select criteria in early years and more complex cases and more experienced operational techniques used in the late years. It worth to note that no death occurred in all the 104 patients who underwent PCI in 1996–1999, this may mainly because more simple coronary artery lesion was selected in the preliminary stage. The rate of stroke in PCI group were 1%, 0.9% and 2.1% separately in the three time periods, which in lower than those in the non-PCI groups. But it is hard to understand that there was no hemorrhagic stroke. This may come from the truth that those cases with hemorrhagic stroke were not transferred to our center. In fact, it is recorded in China that the incidence of intra cerebral hemorrhagic was 0.9%∼1.0%. The rates of GIB in PCI group were between 0∼0.5% in the three time periods, which were lower than that in the conservative group.

### Predictors of Mortality at 30 Days and 1 Year

Firstly, our study shows age to be a positive predictor for both 30-day and one-year mortality, which was in line with other reports [7], [28].

Secondly, the study suggests that PCI is a negative predictor for both 30-day and one-year mortality, after adjusting for age and all other factors. The beneficial performance of PCI in respect of mortality was also certified by ROC analysis.

Thirdly, other factors may have impacted the outcomes, as described briefly here starting with gender. The impact of female gender on clinical outcome is controversial [32], [33]. In a trial involved 500 consecutive patients, a seven-year follow-up found no gender difference in 30-day mortality and that gender was not an independent predictor of late mortality [33]. In our study, only a relatively small proportion of the STEMI patients were female (15.7% of those receiving PCI and 23.8% of those receiving conservative therapy). Female gender was significantly associated with a higher 30-day and one-year mortality, which was in contrast with those of PCI Registry from Paris and AMIS Plus Registry [34]. The different result may be explained by the different age, sample, and years involved.

In this series, the incidences of 30-day and one-year mortality were remarkably higher in AF and hypertensive patients on a univariate analysis, but a multivariate regression analysis did not demonstrate such an independent predictive value. The result of AF was in accordance with Lin, but that of hypertension was not found in Lazzeri’s study, in which no difference between hypertensive and non-hypertensive patients were detected in the in-hospital mortality and Kaplan-Meier survival rates [35], [36].

In accordance with Tsujita, baseline anemia was present in 8.9%, 24.6% and 38.9% of patients in the three age groups (age<65, age = 65–74, age≥75), respectively. The univariate analysis show a more than twofold increase in both 30-day and one-year mortality in patients with anemia, but the multivariate analysis did not show the same association. This may be due to gender difference, because the mortality in Tsujita’s report was significantly higher only in men with baseline anemia (4.6% at 30 days; 8.9% at one year) but not in women [37].

The impact of diabetes on clinical outcomes after PCI has been debated extensively. [38]–[40]. Generally contrary to these studies, we found that DM was not a positive predictor of both 30-day and one-year mortality after multivariate analysis, although it was so in univariate analysis. This is partly consistent with Yip, who found that DM was a predictor for 30-day mortality only on a univariate logistic regression analysis [16]. The different results may come from the different age, gender and other variables involved in these trials; for example, only elderly (75–84 years) patients were included in the MONICA/KORA study [38].

In this study, cardiogenic shock was the strongest predictor of both 30-day and one-year mortality, which is consistent with that reported by other authors [29], [41]. As for CHF, several trials have reported that PCI in patients with ischemic HF and angina resulted in increased mortality, ranging from 5% to 30%, and that AMI patients with CHF have a poor prognosis, elderly or otherwise, which was confirmed by our study [16], [29], [42].

The impact of VT/VF on outcomes in patients with STEMI was analyzed by Mehta who reported that VT/VF occurred in 5.7% of STEMI patients and that 90-day mortality was worse in patients with vs. those without VT/VF [43]. Our data showed VT/VF as the second strongest predictor for both 30-day and one-year mortality (HR, 2.67 and 3.02, respectively).

As reported in other trials [29], our data implied CRF was a strong predictor for 30-days and one-year mortality in this study. This is almost the same as in the HORIZONS-AMI study, in which patients with CRF had higher rates of death than those without (18.7% vs. 4.4%) and baseline creatinine was an independent predictor for death at three years [44].

The effect of pneumonia on clinical outcomes after PCI has been rarely discussed. Our data showed that 3.1%∼20.5% of STEMI patients in the different age groups had pneumonia and that pneumonia was a positive predictor for one-year mortality (HR = 1.446), but not for 30-day mortality. This was partly in accordance with a study in Florida in which 4.6% of STEMI patients had pneumonia and these people were three times more likely (19.6% vs 6.5%) to die before discharge [45].

We also found that CVD was a powerful independent predictor of in-hospital cardiovascular mortality, as pointed out also by Ergelen [46].

In our series, the incidence of GIB was 0.4% and it was an independent predictor of mortality. The same result was seen in the NIS database, in which the incidence of GIB was 1.04% and the adjusted in-hospital mortality 4.70 [47].

For the first time, we found MOF to be another strong predictor for mortality, perhaps because both HF and CRF are predictors in this series.

As for OMI, the negative predicting effect for survival had been reported by different studies [48], [49]. We found, however, that OMI was a negative predictor for one-year mortality (HR:0.616). This may be explained by the different patient profile, because its independent predicting effect for in-hospital death was observed only in AMI patients with cardiogenic shock [49].

We would like to point out that this study is not a randomized clinical trial but an observational cohort study. Despite using multivariable analysis to adjust for the many possible confounders that may be correlated with study outcomes, we cannot exclude the possibility of residual confounding. Furthermore, the rate of re-infarction, the effects of hyperlipidemia, angiographic profile and smoking could not be assessed because they were not recorded. Nevertheless, these consecutive unselected patients do represent real world practice, whereas patients enrolled in clinical trials are carefully selected. The significant lower short-term and long-term mortality of PCI performed 12 hours to 28 days after STEMI in all ages of patients including the elderly strongly indicated a better efficacy of PCI. Above all, the present study is the largest study pertaining specifically to the elder patients undergoing delayed PCI.

### Conclusions

The elderly have more comorbidity and higher rates of mortality, mandating thorough clinical evaluation before acceptance for PCI. PCI after 12 hours to 28 days on a broad age scope (i.e. including the elderly) with STEMI is significantly more effective than conservative therapy, with acceptable short-term and long-term mortality. The data implies that age or time delay alone should not preclude aggressive treatment in patients with STEMI.
